# Juvenile idiopathic arthritis: magnetic resonance imaging of the clinically unaffected knee

**DOI:** 10.1007/s00247-017-4059-7

**Published:** 2018-01-06

**Authors:** E. Charlotte van Gulik, Mendy M. Welsink-Karssies, J. Merlijn van den Berg, Dieneke Schonenberg-Meinema, Koert M. Dolman, Anouk M. Barendregt, Charlotte M. Nusman, Mario Maas, Taco W. Kuijpers, Robert Hemke

**Affiliations:** 1 Department of Radiology and Nuclear Medicine, Academic Medical Center, University of Amsterdam, Department of Radiology (G1-213), Meibergdreef 9, 1105AZ, Amsterdam, the Netherlands; 2 Department of Pediatric Hematology, Immunology, Rheumatology and Infectious Diseases, Emma Children’s Hospital AMC, University of Amsterdam, Amsterdam, the Netherlands; 3 Department of Pediatric Rheumatology, Onze Lieve Vrouwe Gasthuis West, Amsterdam, the Netherlands; 4 Department of Pediatric Rheumatology, Reade, Amsterdam, the Netherlands; 5 Department of Pediatric Rheumatology, Onze Lieve Vrouwe Gasthuis Oost, Amsterdam, the Netherlands

**Keywords:** Clinical scoring, Juvenile idiopathic arthritis, Knee, Magnetic resonance imaging, Subclinical disease activity

## Abstract

**Background:**

Synovial thickening detected on magnetic resonance imaging (MRI) is present in a significant number of children with clinically inactive juvenile idiopathic arthritis (JIA).

**Objective:**

To evaluate patient characteristics and disease activity parameters in a cohort of children with clinically inactive JIA, both with and without synovial thickening, in order to clarify the observed discrepancy between clinical and MRI assessments.

**Materials and methods:**

We prospectively enrolled 52 clinically inactive JIA patients (median age 13.3 years, 63.5% girls) who underwent MRI of the knee as major target joint in JIA. Children were divided into two groups based on MRI outcome: group 1, with synovial thickening on MRI; and group 2, with no synovial thickening on MRI. We used the Juvenile Arthritis MRI Scoring system to evaluate synovial thickness. We compared patient characteristics and disease activity parameters between the groups.

**Results:**

Synovial thickening on MRI was present in 18 clinically inactive patients (group 1, 34.6%). The age was significantly lower for the patients in group 1 (median 10.7 versus 14.4, *P*=0.008). No significant differences were observed in any of the other patient characteristics nor the disease activity parameters tested.

**Conclusion:**

Synovial thickening on MRI was present in nearly 35% of the children with clinically inactive JIA. Children with synovial thickening on MRI were significantly younger than those without. This might indicate that younger patients are at risk of subclinical disease activity and under-treatment, although the exact clinical relevance of synovial thickening on MRI has not been determined.

## Introduction

Juvenile idiopathic arthritis (JIA) is the most common cause of chronic joint inflammation in childhood and represents one of the leading causes of pediatric acquired disability [1]. It encompasses a heterogeneous group of diseases in which clinical presentation, disease course and clinical outcome vary. In JIA, the general aim is to improve long-term outcome by early detection and treatment of disease activity and to identify children who are at risk for joint destruction and poor functional outcome [2, 3]. This requires accurate and sensitive determination of synovitis, which is the hallmark of disease activity. The introduction of the American College of Rheumatology criteria for disease activity [4] and the Wallace criteria for clinical inactive disease (clinically inactive disease) [5] have improved the interpretation of the clinical assessment; however the reliability of the clinical assessment remains unclear [6, 7].

MRI is a frequently used imaging modality in disease activity assessment of JIA. On MRI, thickened synovium (≥2 mm), which enhances after contrast administration, is considered to represent ongoing inflammation of the synovial membrane (synovitis) because it has been shown to be responsive to treatment [8, 9]. Also, as recently published by Hemke et al. [10], the synovial thickness in knees of healthy children does not exceed 1.8 mm. When clinical assessment shows no signs of inflammation but synovial thickening is observed on MRI, it is commonly interpreted as subclinical synovitis. In JIA, it was previously described that synovial thickening on MRI is present in up to 50% of the JIA patients who are considered to be clinically inactive. Therefore, it is stated that MRI is more sensitive than clinical assessment in detecting disease activity [7, 11, 12].

However, another explanation for the observed synovial thickening on MRI is a persistent synovial change after chronic inflammation. Because of this controversy, the clinical relevance of synovial thickening on MRI remains unclear. If synovial thickening on MRI in clinically inactive children represents ongoing disease activity, these children might receive insufficient treatment and could potentially benefit from adapted treatment regimens, in order to prevent irreversible destructive changes. If synovial thickening represents persistent synovial alteration after inflammation, treatment regimens would not have to be adapted or could even be stopped.

To optimize the clinical decision-making efficacy of MRI in JIA patients with clinically inactive disease, underlying reasons for the observed discrepancy between clinical assessment and MRI findings need to be better understood. Therefore we evaluated patient characteristics and disease activity parameters in a cohort of clinically inactive JIA patients with and without subclinical signs of synovitis on MRI.

## Materials and methods

### Patient selection

We retrospectively identified children in the Amsterdam JIA Cohort Studies. The setup of this cohort in 2008 was to prospectively enroll children suspected of having JIA and to collect a predefined set of variables at the time a JIA patient visited one of the outpatient clinics and was scheduled for an MRI. We included children visiting a tertiary pediatric rheumatology center (Academic Medical Centre [AMC], Amsterdam) or a non-academic pediatric rheumatology center (Reade and Onze Lieve Vrouwe Gasthuis, both Amsterdam, the Netherlands) who were scheduled for an MRI at the AMC to assess disease activity between December 2008 and December 2014.

All children involved in this study underwent clinical and laboratory assessment, followed by contrast-enhanced MRI of the knee. The MRI was requested as part of standard care. When multiple MRIs were performed in the same child at a time of clinically inactive disease, we chose the first MRI. The knee was chosen as the target joint because it is the most commonly involved joint in JIA [13]. Inclusion criteria were: (1) children fulfilling the International League of Associations for Rheumatology criteria for JIA, defined as arthritis of unknown etiology that begins before age 16 and persists for at least 6 weeks [14]; (2) children with clinically inactive disease according to the Wallace criteria [5], defined as having no joints with active arthritis; no fever, rash, serositis, splenomegaly or generalized lymphadenopathy attributable to JIA; no active uveitis; erythrocyte sedimentation rate or C-reactive protein levels within normal limits; and a best possible score for the physician’s global assessment of disease activity on the scale used; and (3) a history of clinically evident arthritis of the knee subject to MRI.

**Fig. 1:**
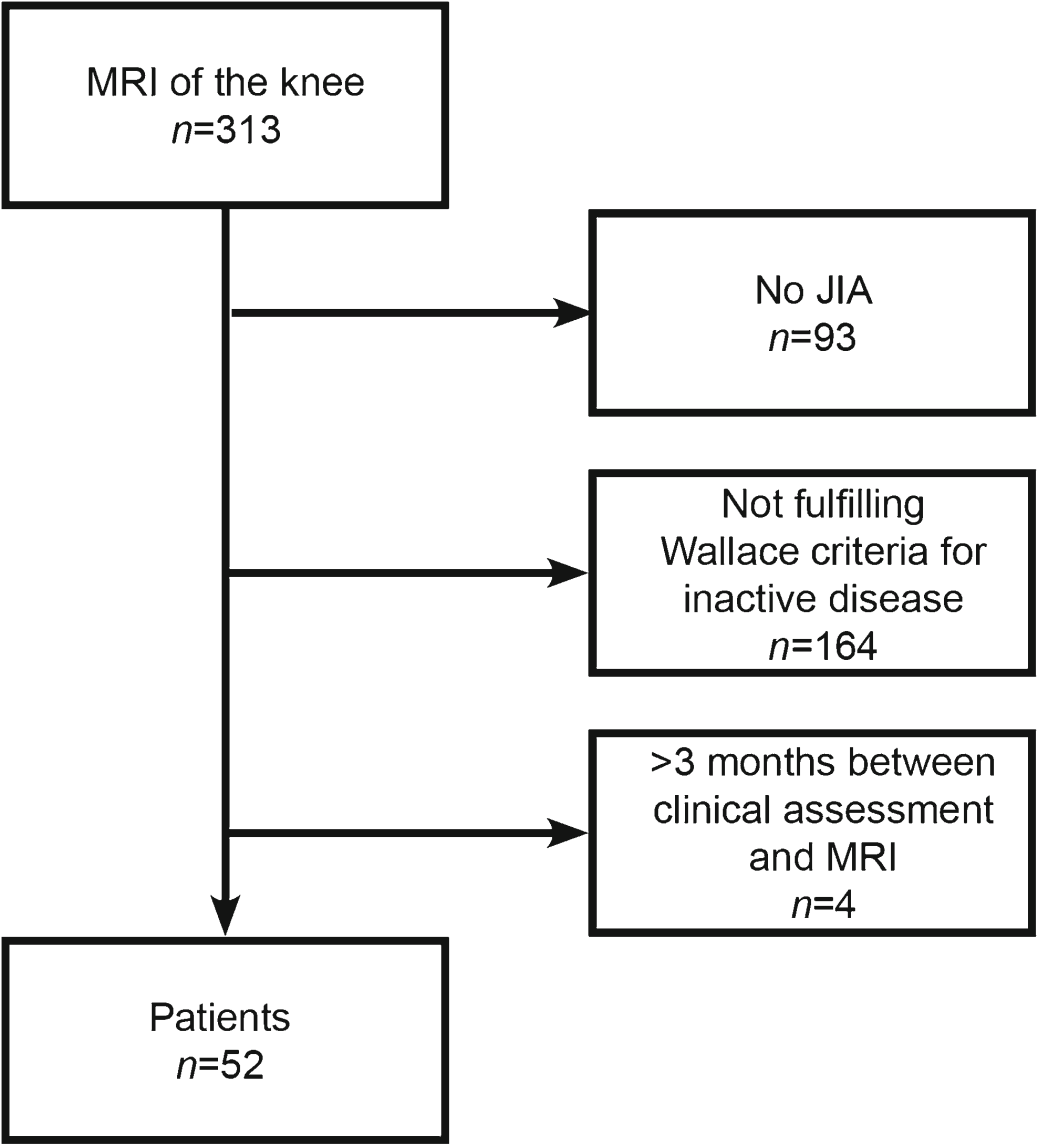
Flowchart of patient selection. Wallace criteria are defined as having no joints with active arthritis; no fever, rash, serositis, splenomegaly or generalized lymphadenopathy attributable to JIA; no active uveitis; erythrocyte sedimentation rate or C-reactive protein levels within normal limits, and a best possible score for the physician’s global assessment of disease activity on the scale used. *JIA* juvenile idiopathic arthritis, *MRI* magnetic resonance imaging

Children were excluded if (1) the knee undergoing MRI was active according to the definition of the American College of Rheumatology [5], defined as a joint with swelling not caused by bony enlargement or, if no swelling is present, limitation of motion accompanied by either pain on motion and/or tenderness. (An isolated finding of pain on motion, tenderness, or limitation of motion on the joint examination could be present only if explained by either prior damage attributable to arthritis now considered as inactive or caused by non-rheumatologic reasons, such as trauma); and (2) the MRI was not performed within 3 months after the clinical assessment. A flowchart of patient selection is depicted in Fig. 1. The local ethics committee waived the requirement for informed consent for this study.

### Clinical assessment

The clinical assessment was performed by one of our experienced pediatric rheumatologists and consisted of the following: an 84-joint count defining presence of swelling, pain on motion/tenderness and limited range of motion. The physician’s global assessment of disease activity, patient’s global assessment of well-being and patient’s assessment of pain were measured on a visual analog scale (VAS; range 0–100 mm, with 0 being the best score). A physician’s global assessment <10 was interpreted as inactive. Consequently, the physician noted the presence of the above-mentioned symptoms attributable to JIA as defined by Wallace and calculated the Juvenile Arthritis Disease Activity Score [15]. We used the Dutch version of the childhood health assessment questionnaire to evaluate functional ability of the patients [16]. General and immunology laboratory tests included erythrocyte sedimentation rate, C-reactive protein, immunoglobulin M (IgM) rheumatoid factor, antinuclear antibody, human leukocyte antigen B-27 and anti-cyclic citrullinated peptides. Medication use was noted.

### Magnetic resonance imaging protocol

MRI of the target knee was performed using an open-bore 1.0-Tesla (T) MRI scanner with a dedicated knee coil (Panorama HFO; Philips Medical Systems, Best, The Netherlands). The children were placed in supine position with the knee centrally in the magnetic field. Details on the MRI sequences are summarized in Table 1.

**Table 1:** Magnetic resonance imaging acquisitions

Sequence	Plane	FS	Gd	TR (ms)	TE (ms)	ST (mm)	Spacing	FOV (mm)	Matrix
T2 SPIR	Sag	+	–	2800–4327	50	4	0.4	150 × 150	300 × 423
T1 TSE	Sag	–	–	515–591	10	4	0.4	150 × 150	332 × 236
T2 SPIR	Cor	+	–	2700–4500	50–60	4	0.4	150 × 150	300 × 247
T2 SPIR	Ax	+	–	2800–4500	50	4	0.4	150 × 150	300 × 270
T1 SPIR	Ax	+	+	588–591	10	4	0.4	150 × 150	272 × 192
T1 TSE	Sag	–	+	518–592	10	4	0.4	150 × 150	332 × 236

*Ax* axial, *Cor* coronal, *FOV* field of view, *FS* fat saturation (+: yes; −: no), *Gd* intravenous injection of gadolinium contrast (−: before Gd injection; +: after Gd injection); *Sag* sagittal, *SPIR* spectral presaturation inversion recovery, *ST* slice thickness, *TE* echo time, *TR* repetition time, *TSE* turbo spin echo

Post-contrast images were obtained within the early phase (within 5 minutes) after intravenous injection of a gadolinium-containing contrast agent (0.1 mmol/kg of body weight, gadobutrol; Bayer Healthcare, Berlin, Germany).

### Image analysis

**Fig. 2:**
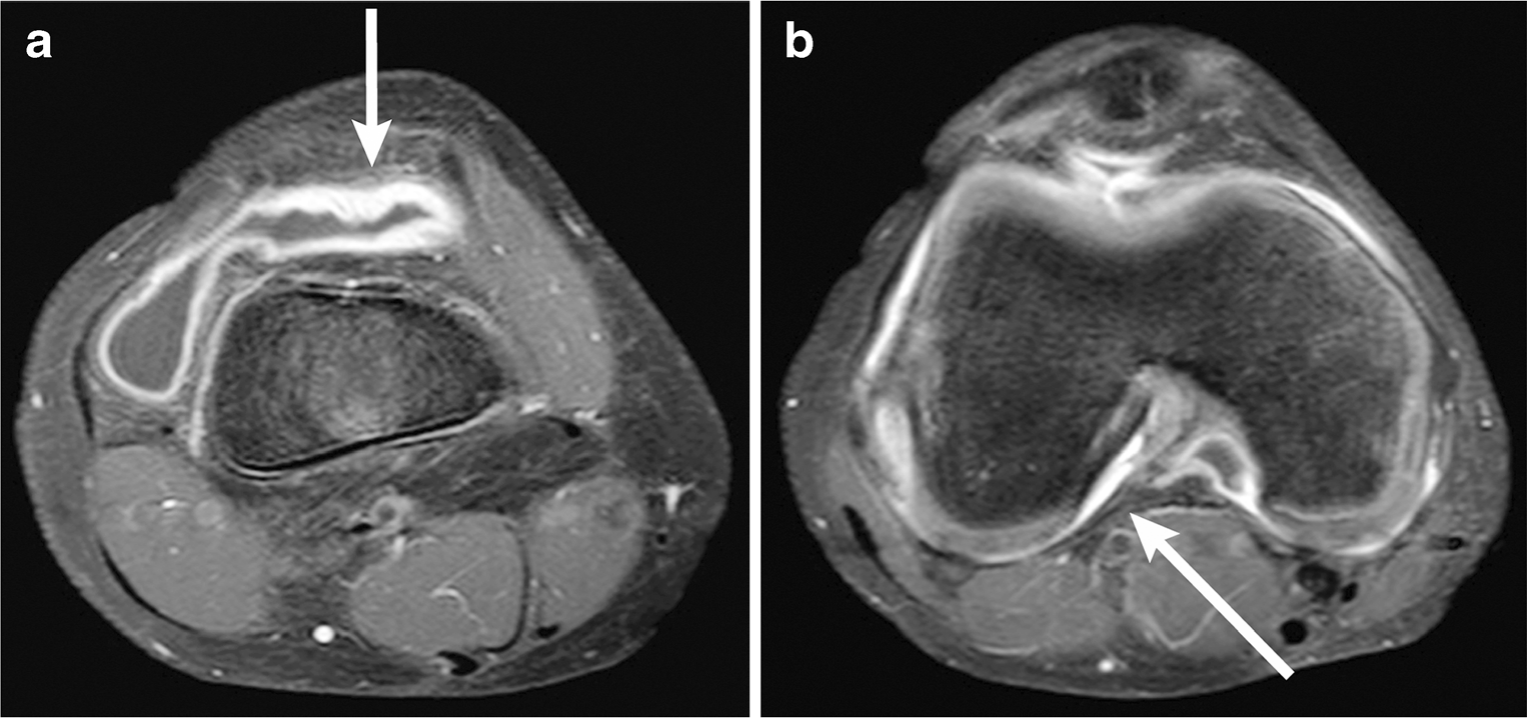
Two examples of how synovial thickness can be measured. Axial T1-weighted fat-saturated MRI images at the suprapatellar region (**a**) and at the cruciate ligaments (**b**) of a 10-year old male. In the suprapatellar region (**a**) contrast shows a synovial thickness of at most 4.1 mm (*arrow*), corresponding to a JAMRIS score of 2 (≥ 4 mm) on this location. At the cruciate ligaments (**b**) the enhanced synovium is measured at 2.0 mm (*arrow*), corresponding to a JAMRIS score of 1 (≥2 - 4 mm)

The MRI dataset was scored by one reader (R.H., 6 years of experience in musculoskeletal radiology) who was blinded to the clinical disease state of the patients. In order to quantify disease activity on MRI, synovial thickening was scored using the validated and reliable Juvenile Arthritis MRI Scoring system (JAMRIS) [17]. In the JAMRIS system, synovial thickening is defined as an area of increased signal of the synovial compartment on MRI that shows a thickness greater than the width of the normal synovium (normal <2 mm) [10]. The synovium is scored at six locations in the knee joint: the patellofemoral joint, the suprapatellar recess, the infrapatellar fat pad, the cruciate ligaments, the medial posterior condyle and the lateral posterior condyle. Per location a score of 0, 1 or 2 can be given, corresponding to a synovial thickness of 0–2 mm, ≥2–4, mm and ≥4 mm, respectively, resulting in a maximum score of 12 [17]. Two examples of a JAMRIS measurement are depicted in Fig. 2. A JAMRIS synovial thickening score of ≥1 is considered positive and interpreted as synovitis.

### Statistical analysis

We report on descriptive statistics of patient characteristics and disease activity parameters. Because data were not normally distributed, we used non-parametric tests to test for differences between patients with and without synovial thickening on MRI. We used chi-square and Fisher exact tests to analyze differences between groups when data were categorical (respectively binary and nominal/ordinal data). When data were continuous we used the Mann–Whitney *U* test to analyze differences between groups. All tests assumed a two-tailed probability and a *P*-value of <0.05 was considered significant. Because of the exploratory nature of the analyses, we did not perform correction for multiple testing [18, 19]. We used binary logistic regression to identify significant predictors or signs of synovitis on MRI. This is reported with odds ratios and a 95% confidence interval. We analyzed all data using SPSS software version 22 (IBM Corp., Armonk, NY).

## Results

### Patient characteristics

**Fig. 3:**
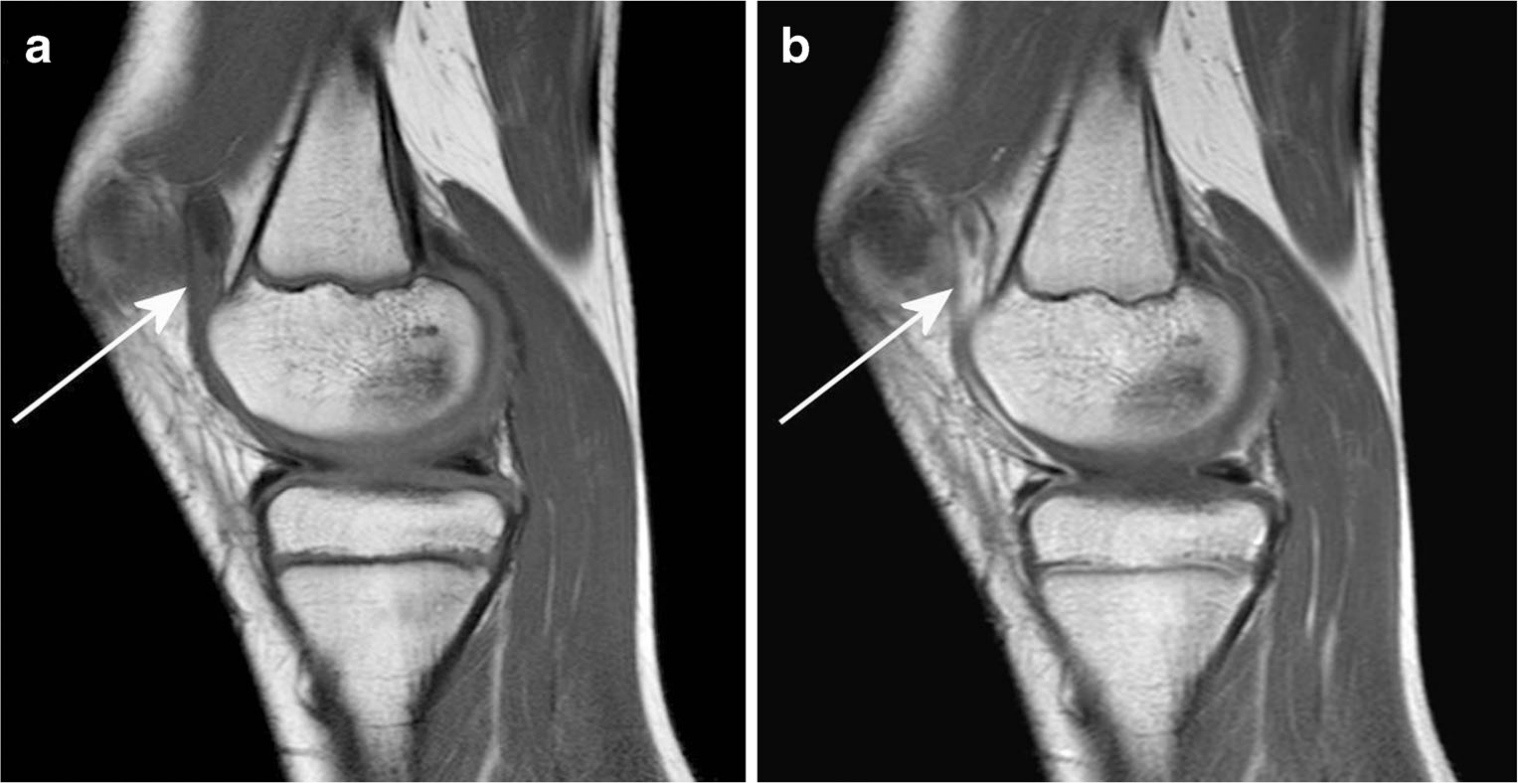
Sagittal T1-weighted MRI sequence (TR/TE 518/10 ms) of the right knee in a 10-year-old girl with clinically inactive juvenile idiopathic arthritis of the oligoarticular subtype. **a, b** Pre-(**a**) and post-contrast (**b**) images show enhancing and thickened synovium at the patellofemoral region (*arrow*)

Of the 313 children who underwent an MRI of the knee, a total of 261 were excluded because of (1) a diagnosis other than JIA during follow-up (*n*=93), (2) not fulfilling the Wallace criteria for inactive disease (*n*=164), or (3) there was a time lapse of more than 3 months between clinical assessment and imaging (*n*=4). This resulted in a total of 52 included JIA patients. These children were divided into two groups based on the MRI outcome: group 1 consisted of 18 patients (34.6%, 95% confidence interval 22.3–49.2%) with clinically inactive disease and synovial thickening on MRI (JAMRIS synovial hypertrophy score of ≥1), and group 2 consisted of 34 patients (65.4%) with clinically inactive disease without synovial thickening on MRI (JAMRIS synovial hypertrophy score of 0). An example of a child with clinically inactive disease and synovial thickening on MRI is shown in Fig. 3. Patient characteristics and disease activity parameters are summarized in Table 2.

**Table 2:** Patient characteristics of children with clinically inactive juvenile idiopathic arthritis with and without synovial thickening on MRI

Variable		Total *n*=52	Group 1 JAMRIS ≥1 *n*=18	Group 2 JAMRIS = 0 *n*=34	*P*-value
Female gender, *n* (%)		33 (63.5)	10 (19.2)	23 (44.2)	0.389
Age, years		13.3 (10.4–15.7)	10.7 (9.3–13.6)	14.4 (12.1–16.3)	0.008
Waiting period, days^a^		35 (27.5–53.0)	36 (23.75–45.5)	35 (28.0–54.0)	0.855
JIA parameters					
Age at disease onset		9.4 (6.0–12.3)	7.9 (5.7–10.9)	10.6 (6.1–13.3)	0.260
Disease duration (subjective)^b^		3.9 (2.4–6.6)	3.1 (1.8–7.0)	4.3 (2.9–6.5)	0.178
Disease duration (objective)^c^		2.8 (1.1–5.6)	2.1 (1.0–5.3)	3.2 (1.7–5.8)	0.237
Duration of inactivity (days)		198 (34–470.5)	192.5 (28–275)	226 (34–571)	0.256
Uveitis in patient history, *n* (%)		3 (5.8)	0 (0.0)	3 (8.8)	
Disease activity parameters					
CHAQ		0.3 (0.0–0.8)	0.3 (0.0–0.8)	0.3 (0.0–0.9)	0.707
JADAS-10		1.4 (0.3–3.2)	1.0 (0.3–4.1)	1.5 (0.4–2.9)	0.400
Physician’s VAS		1.0 (0.0–4.0)	0.0 (0.0–3.3)	2.0 (0.0–4.5)	
Patient’s VAS					
- Pain		12.0 (0.0–32.0)	9.0 (0.0–45.0)	14.5 (0.0–25.3)	0.751
- Global		5.0 (0.0–22.0)	5.0 (0.0–49.0)	6.5 (0.0–20.5)	0.980
JAMRIS^d^		0.0 (0.0–1.0)	1.0 (1.0–3.3)	0 (0.0)	
Laboratory results					
ANA	Positive, *n* (%)	7 (13.5)	3 (16.7)	4 (11.8)	
	Negative, *n* (%)	45 (86.5)	15 (83)	30 (78.9)	
HLA-B27	Positive, *n* (%)	8 (15.4)	4 (22.2)	4 (11.8)	
	Negative, *n* (%)	38 (73.1)	13 (72.2)	25 (65.8)	
IgM RF	Positive, *n* (%)	0 (0.0)	0 (0.0)	0 (0.0)	
	Negative, *n* (%)	49 (94.2)	16 (88.9)	33 (97.1)	
Anti-CCP	Positive, *n* (%)	0 (0.0)	0 (0.0)	0 (0.0)	
	Negative, *n* (%)	50 (96.2)	16 (88.9)	34 (100)	
Medication use					
None		7 (13.5)	2 (11.1)	5 (14.7)	
NSAID		3 (5.8)	0 (0.0)	3 (8.8)	
Methotrexate^e^		31 (59.6)	11 (61.1)	20 (58.8)	
Sulfasalazine^e^		5 (9.6)	3 (16.7)	2 (5.9)	
Etanercept		2 (3.8)	2 (11.1)	0 (0.0)	
Etanercept + Methotrexate^d^		4 (7.7)	0 (0.0)	4 (11.8)	

*ANA* antinuclear antibody, *Anti-CCP* anti-cyclic citrullinated peptides, *CHAQ* childhood health assessment questionnaire, *HLA-B27* human leukocyte antigen B-27, *IgM RF* immunoglobulin M rheumatoid factor, *JADAS* juvenile arthritis disease activity score, *JAMRIS* juvenile arthritis MRI scoring system, *JIA* juvenile idiopathic arthritis, *NSAID* nonsteroidal anti-inflammatory drug, *VAS* visual analog scale

^a^Number of days between the date of clinical assessment and the date of MRI

^b^Number of years between disease onset as experienced by the patient/parents and date of the clinical assessment

^c^Number of years between date of diagnosis and date of the clinical assessment

^d^JAMRIS ≥1 is defined as a synovial thickness ≥2 mm on at least one location in the knee

^e^Some children used an additional nonsteroidal anti-inflammatory drugs

Of the 52 included patients, 33 (63.5%) were girls. The median age was 13.3 years (interquartile range [IQR] 10.4–15.7). The subtypes as defined by the International League of Associations for Rheumatology were represented as follows: 22 (42.3%) oligoarthritis, 23 (44.2%) rheumatoid factor-negative polyarthritis, 4 (7.7%) enthesitis-related arthritis, 1 (1.9%) systemic arthritis, 1 (1.9%) psoriatic arthritis and 1 (1.9%) undifferentiated JIA. Medication use was as follows: 10 children used no disease-modifying antirheumatic drugs (DMARDs) while the majority of 42 patients were still on anti-inflammatory medication using one or more DMARDs. Treatment, as prescribed by the pediatric rheumatologist, was not adjusted between clinical examination and the time of MRI.

The indications to order an MRI were as follows: 28 (53.8%) to determine whether medication could be stopped, 16 (30.8%) to exclude any doubt in a clinically inactive joint with some post-inflammatory pain and swelling, and 8 (15.4%) when there was a discrepancy between the physician’s view (clinically inactive disease) and the view of the patient or parents. Regarding these indications, there was no statistically significant difference between the groups.

### Comparison between children with and without synovial thickening on MRI

The children in group 1 were significantly younger (median age 10.7 years in group 1 vs. median age 14.4 years in group 2, *P*=0.008). As an independent predictor, an increase in age at the clinical visit was associated with reduced odds of having a JAMRIS ≥1 (odds ratio 0.816, 95% confidence interval 0.68 to 0.98, *P*=0.029). The percentage of children with rheumatoid-factor-negative polyarticular JIA was significantly lower in group 1 (22% vs. 56% in group 2, *P*=0.038). We observed no significant differences between the groups regarding the other patient characteristics and disease activity parameters. Additionally, we observed no significant differences in enhancement between patients in each group.

## Discussion

Our study is the first to evaluate differences in patient characteristics and disease activity parameters in a group of children with clinically inactive JIA with and without synovial thickening. Children with clinically inactive JIA and synovial thickening upon MRI were significantly younger compared to the clinically inactive JIA patients without synovial thickening on MRI. Our results indicate that younger JIA patients might benefit from additional and more regular monitoring of disease activity with the use of MRI.

A significant portion of the children with clinically inactive JIA showed synovial thickening on MRI (34.6%). These results are in line with previous studies in JIA patients addressing the discrepancy between the clinical assessment and MRI findings [7, 20, 21]. Because longitudinal studies evaluating synovial thickening on MRI in children with clinically inactive JIA are absent, it remains difficult to determine the cause(s) of the observed synovial thickening on MRI. Although the possibility exists that the observed synovial thickening is benign — remnants of active disease in the past — it might also reflect ongoing disease activity. If so, these children might receive insufficient treatment and could be at risk for joint destruction and an unfavorable outcome.

In this study the children in whom the clinical examination and the MRI were discrepant were younger. Why this discrepancy occurs is unknown. We hypothesize that younger children are less comprehensive in expressing their complaints. Additionally, on many occasions, the parent takes the lead in the conversation with the pediatric rheumatologist. Both situations could make it difficult for the pediatric rheumatologist to relate physical examination to the actual complaints and increase the risk of underreporting disease activity.

Limitations of this study are the variations among children in the length of time between clinical examination and MRI, and the differences in received treatment. This study followed general clinical practice; therefore the timeframe between clinical visit and MRI was not standardized. Because the children in this study had inactive or remitting disease at the time of clinical examination as well as at the time of MRI, the effect of a prolonged waiting period is considered minimal. The limit of 3 months is meant as a reflection of clinical practice, based on the period in which the child returns to the pediatric rheumatologist and treatment alterations are being made.

The applied treatment regimens differed among patients, ranging from no medication to the combination of biologicals with non-biologicals. Treatments of individual children did not alter in the period between clinical visit and MRI because the MRI was mainly requested as an aid in treatment decision-making. It is therefore not expected that the different treatments would have an effect on the MRI outcome.

In rheumatoid arthritis, MRI is considered to be of great value in detecting and predicting disease activity [22–25]. Despite the similarities between rheumatoid arthritis and JIA, the results obtained in adults with rheumatoid arthritis cannot simply be translated to the JIA population. For example in people with rheumatoid arthritis, bone marrow edema is considered to represent disease activity and to predict disease progression [26, 27]. The clinical relevance of bone marrow edema in children with JIA remains unclear and because of its high prevalence in healthy children it might be considered a characteristic of normal bone maturation [8, 28]. The questions rise, what exactly is measured on MRI and should the pediatric rheumatologist alter the treatment plan solely on signs of inflammation still visible on MRI? MRI has been more widely used in the last few years in JIA; however the predictive value of synovial thickening for the development of a worse outcome remains to be defined and validated because no prospective studies have been performed. Critical evaluation of subclinical disease is therefore warranted, as we must at all times ensure not to treat “MRI-tis.”

When considering synovial thickening as a remnant of disease activity, we hypothesize that when duration of inactivity increases, the chance of finding synovial thickening on MRI decreases. Because of the wide range of duration of inactivity, this cannot be concluded from this study. This should be taken into account in future studies because it would contribute to understanding the true meaning of synovial thickening.

To determine the role and value of MRI in clinical practice in children with JIA, future longitudinal and prospective research needs to be standardized to evaluate whether children with clinically inactive JIA and synovial thickening are at risk for ongoing disease activity. Ideally, a baseline MRI should be made and thereafter children with clinically inactive JIA should be randomly divided into homogeneous groups with or without treatment adjustments, thus optimizing the comparability between groups in order to analyze the treatment outcome.

Further development of easy interpretable imaging techniques might support the pediatric rheumatologist in the joint assessment, especially if the clinical assessment is inconclusive. Currently the clinical assessment and MRI complement each other and are both needed in the accurate determination of disease activity in children with JIA. In the meantime, the observed discrepancy between the clinical assessment and MRI should be interpreted with care.

## Conclusion

In more than one-third of children with clinically inactive disease in our cohort, MRI of the knee showed synovial thickening. This might indicate prolonged, subclinical inflammation of the joint or could represent a remnant of disease activity. Because both explanations have different implications for treatment decision-making, it is important to unravel the pathophysiology of synovial thickening on MRI in children with clinically inactive juvenile idiopathic arthritis.
